# Supplementary material for: Degraded RNA from Human Anterior Cruciate Ligaments Yields Valid Gene Expression Profiles
Source: Int J Mol Sci. 2023 Jan 18;24(3):1895. doi: 10.3390/ijms24031895 (PMC9916516; doi:10.3390/ijms24031895)
Supplement: Supplementary file 1 [file ijms-24-01895-s001.zip › ijms-2139982-supplementary.pdf]

Accession	Gene	Gene product
NM_006988	ADAMTS1	ADAM metallopeptidase with thrombospondin type 1 motif, 1
NM_139025	ADAMTS13	ADAM metallopeptidase with thrombospondin type 1 motif, 13
NM_007037	ADAMTS8	ADAM metallopeptidase with thrombospondin type 1 motif, 8
NM_000610	CD44	CD44 molecule (Indian blood group)
NM_004360	CDH1	Cadherin 1, type 1, E-cadherin (epithelial)
NM_003278	CLEC3B	C-type lectin domain family 3, member B
NM_001843	CNTN1	Contactin 1
NM_080629	COL11A	Collagen, type XI, alpha 1
NM_004370	COL12A	Collagen, type XII, alpha 1
NM_021110	COL14A	Collagen, type XIV, alpha 1
NM_001855	COL15A	Collagen, type XV, alpha 1
NM_001856	COL16A	Collagen, type XVI, alpha 1
NM_000088	COL1A1	Collagen, type I, alpha 1
NM_001846	COL4A2	Collagen, type IV, alpha 2
NM_000093	COL5A1	Collagen, type V, alpha 1
NM_001848	COL6A1	Collagen, type VI, alpha 1
NM_001849	COL6A2	Collagen, type VI, alpha 2
NM_000094	COL7A1	Collagen, type VII, alpha 1
NM_001850	COL8A1	Collagen, type VIII, alpha 1
NM_001901	CTGF	Connective tissue growth factor
NM_001903	CTNNA1	Catenin (cadherin-associated protein), alpha 1, 102kDa
NM_001904	CTNNB1	Catenin (cadherin-associated protein), beta 1, 88kDa
NM_001331	CTNND1	Catenin (cadherin-associated protein), delta 1
NM_001332	CTNND2	Catenin (cadherin-associated protein), delta 2 (neural plakophilin-related arm-repeat protein)
NM_004425	ECM1	Extracellular matrix protein 1
NM_002026	FN1	Fibronectin 1
NM_001523	HAS1	Hyaluronan synthase 1
NM_000201	ICAM1	Intercellular adhesion molecule 1
NM_181501	ITGA1	Integrin, alpha 1
NM_002203	ITGA2	Integrin, alpha 2 (CD49B, alpha 2 subunit of VLA-2 receptor)
NM_002204	ITGA3	Integrin, alpha 3 (antigen CD49C, alpha 3 subunit of VLA-3 receptor)
NM_000885	ITGA4	Integrin, alpha 4 (antigen CD49D, alpha 4 subunit of VLA-4 receptor)

NM_002205	ITGA5	Integrin, alpha 5 (fibronectin receptor, alpha polypeptide)
NM_000210	ITGA6	Integrin, alpha 6
NM_002206	ITGA7	Integrin, alpha 7
NM_003638	ITGA8	Integrin, alpha 8
NM_002209	ITGAL	Integrin, alpha L (antigen CD11A (p180), lymphocyte function-associated antigen 1; alpha polypeptide)
NM_000632	ITGAM	Integrin, alpha M (complement component 3 receptor 3 subunit)
NM_002210	ITGAV	Integrin, alpha V (vitronectin receptor, alpha polypeptide, antigen CD51)
NM_002211	ITGB1	Integrin, beta 1 (fibronectin receptor, beta polypeptide, antigen CD29 includes MDF2, MSK12)
NM_000211	ITGB2	Integrin, beta 2 (complement component 3 receptor 3 and 4 subunit)
NM_000212	ITGB3	Integrin, beta 3 (platelet glycoprotein IIIa, antigen CD61)¬†
NM_000213	ITGB4	Integrin, beta 4¬†
NM_002213	ITGB5	Integrin, beta 5
NM_000216	KAL1	Kallmann syndrome 1 sequence
NM_005559	LAMA1	Laminin, alpha 1
NM_000426	LAMA2	Laminin, alpha 2
NM_000227	LAMA3	Laminin, alpha 3
NM_002291	LAMB1	Laminin, beta 1
NM_000228	LAMB3	Laminin, beta 3
NM_002293	LAMC1	Laminin, gamma 1 (formerly LAMB2)
NM_002421	MMP1	Matrix metallopeptidase 1 (interstitial collagenase)
NM_002425	MMP10	Matrix metallopeptidase 10 (stromelysin 2)
NM_005940	MMP11	Matrix metallopeptidase 11 (stromelysin 3)
NM_002426	MMP12	Matrix metallopeptidase 12 (macrophage elastase)
NM_002427	MMP13	Matrix metallopeptidase 13 (collagenase 3)
NM_004995	MMP14	Matrix metallopeptidase 14 (membrane-inserted)
NM_002428	MMP15	Matrix metallopeptidase 15 (membrane-inserted)
NM_005941	MMP16	Matrix metallopeptidase 16 (membrane-inserted)
NM_004530	MMP2	Matrix metallopeptidase 2 (gelatinase A, 72kDa gelatinase, 72kDa type IV collagenase)
NM_002422	MMP3	Matrix metallopeptidase 3 (stromelysin 1, progelatinase)
NM_002423	MMP7	Matrix metallopeptidase 7 (matrilysin, uterine)
NM_002424	MMP8	Matrix metallopeptidase 8 (neutrophil collagenase)
NM_004994	MMP9	Matrix metallopeptidase 9 (gelatinase B, 92kDa gelatinase, 92kDa type IV collagenase)
NM_000615	NCAM1	Neural cell adhesion molecule 1

NM_000442	PECAM1	Platelet/endothelial cell adhesion molecule
NM_000450	SELE	Selectin E
NM_000655	SELL	Selectin L
NM_003005	SELP	Selectin P (granule membrane protein 140kDa, antigen CD62)
NM_003919	SGCE	Sarcoglycan, epsilon
NM_003118	SPARC	Secreted protein, acidic, cysteine-rich (osteonectin)
NM_003119	SPG7	Spastic paraplegia 7 (pure and complicated autosomal recessive)
NM_000582	SPP1	Secreted phosphoprotein 1
NM_000358	TGFBI	Transforming growth factor, beta-induced, 68kDa
NM_003246	THBS1	Thrombospondin 1
NM_003247	THBS2	Thrombospondin 2
NM_007112	THBS3	Thrombospondin 3
NM_003254	TIMP1	TIMP metallopeptidase inhibitor 1
NM_003255	TIMP2	TIMP metallopeptidase inhibitor 2
NM_000362	TIMP3	TIMP metallopeptidase inhibitor 3
NM_002160	TNC	Tenascin C
NM_001078	VCAM1	Vascular cell adhesion molecule 1
NM_004385	VCAN	Versican
NM_000638	VTN	Vitronectin
NM_001101	ACTB	Actin, beta
NM_004048	B2M	Beta-2-microglobulin
NM_002046	GAPDH	Glyceraldehyde-3-phosphate dehydrogenase
NM_000194	HPRT1	Hypoxanthine phosphoribosyltransferase 1
NM_001002	RPLP0	Ribosomal protein, large, P0
	HGDC	Human Genomic DNA Contamination
	RTC	Reverse Transcription Control
	RTC	Reverse Transcription Control
	RTC	Reverse Transcription Control
	PPC	Positive PCR Control
	PPC	Positive PCR Control
	PPC	Positive PCR Control¬†
